# Automatic Polyp Segmentation in Colonoscopy Images Using a Modified Deep Convolutional Encoder-Decoder Architecture

**DOI:** 10.3390/s21165630

**Published:** 2021-08-20

**Authors:** Chin Yii Eu, Tong Boon Tang, Cheng-Hung Lin, Lok Hua Lee, Cheng-Kai Lu

**Affiliations:** 1 Department of Electrical and Electronic Engineering, Universiti Teknologi PETRONAS, Seri Iskandar 32610, Malaysia; chin_19000297@utp.edu.my (C.Y.E.); tongboon.tang@utp.edu.my (T.B.T.); lee.lok_24987@utp.edu.my (L.H.L.); 2 Department of Electrical Engineering and Biomedical Engineering Research Center, Yuan Ze University, Jungli 32003, Taiwan; chlin@saturn.yzu.edu.tw

**Keywords:** colorectal cancer, computer-aided diagnosis (CAD), SegNet Visual Geometry Group-19 (VGG-19), convolutional neural network (CNN), polyp segmentation

## Abstract

Colorectal cancer has become the third most commonly diagnosed form of cancer, and has the second highest fatality rate of cancers worldwide. Currently, optical colonoscopy is the preferred tool of choice for the diagnosis of polyps and to avert colorectal cancer. Colon screening is time-consuming and highly operator dependent. In view of this, a computer-aided diagnosis (CAD) method needs to be developed for the automatic segmentation of polyps in colonoscopy images. This paper proposes a modified SegNet Visual Geometry Group-19 (VGG-19), a form of convolutional neural network, as a CAD method for polyp segmentation. The modifications include skip connections, 5 × 5 convolutional filters, and the concatenation of four dilated convolutions applied in parallel form. The CVC-ClinicDB, CVC-ColonDB, and ETIS-LaribPolypDB databases were used to evaluate the model, and it was found that our proposed polyp segmentation model achieved an accuracy, sensitivity, specificity, precision, mean intersection over union, and dice coefficient of 96.06%, 94.55%, 97.56%, 97.48%, 92.3%, and 95.99%, respectively. These results indicate that our model performs as well as or better than previous schemes in the literature. We believe that this study will offer benefits in terms of the future development of CAD tools for polyp segmentation for colorectal cancer diagnosis and management. In the future, we intend to embed our proposed network into a medical capsule robot for practical usage and try it in a hospital setting with clinicians.

## 1. Introduction

Among all types of cancer, colorectal cancer is the third most commonly diagnosed [1], and has the second highest cancer fatality rate worldwide [2]. It is the most common cancer in men and women in Malaysia, as reported in [3]. Colorectal cancer involves a type of malignant tumour which can be found in the colon or rectum. In the early stages, the tumour (polyp) is an abnormal tissue growth on the intestinal wall. Polyps are initially benign, but can potentially develop into malignant tumours after a certain period [4]. The diagnosis of a polyp at an early stage is vital in clinical routines to prevent it from mutating into cancerous cells, thus regular screening is required to search for polyps. Several methods can be used to detect colorectal cancer, such as a high-sensitivity fecal occult blood tests (FOBT), stool deoxyribonucleic acid (DNA) test, optical colonoscopy, and computed tomographic colonography [5]. Optical colonoscopy is the preferred tool for diagnosing polyps [6]. During the colonoscopy procedure, a flexible endoscope with a tiny video camera at the tip is gradually advanced under direct vision via the anus into the proximal part of the colon. The video camera generates thousands of images of the internal mucosa of the colon for real-time manual analysis by the doctor. When polyps are found, they are resected as part of a histological analysis procedure.

Although optical colonoscopy is preferable, there is still a miss rate of approximately 10–30% for a single colon screening procedure [7]. This is primarily due to the complex structure of the colon and human error [8]. In order to extract information on the polyp from the images of the colon and rectum, a doctor usually screens for hours to seek, observe, and diagnose. This overwhelming workload not only causes physical and mental fatigue for the doctor, but can also be a cause of false diagnosis even by a well-trained and experienced clinician [9]. In addition, diagnosis outcomes show large variations in terms of inter-observer and intra-observer decisions [10]. These errors can be correlated with the medical level of expertise and the disparity in the appearance and nature of the polyps themselves. There is a wide variety in the appearance of polyps, in terms of their shapes, sizes, colour, and texture, and the distraction due to convoluted folds and bends inside the colon complicates the diagnostic task [11].

An effective computer-aided diagnosis (CAD) system should improve diagnostic performance, save time, and be seamlessly integrated into the workflow. With the recent advancements, CAD can improve the accuracy and consistency of radiological diagnosis and ease the work of doctors when decreasing the workload and improving the promptness of medical diagnosis. In a real-time colon screening process, only a small number of video frames contain polyps, while most are not informative. A CAD system can help to ease the process of interpreting this invaluable source of information. CAD systems can generally be divided into traditional handcrafted methods and modern deep learning methods. A handcrafted method is used to extract the intrinsic features of a polyp as determined by the researcher, while a deep learning method enables a computer model to learn the features and use these in decision making by automatically extracting features from raw data. In view of the recent achievements of convolutional neural network (CNN) architectures (a type of deep learning model) in solving image processing problems, this has become the state-of-the-art methodology for detection, classification, and segmentation tasks [12]. Many ensemble methods yield great results due to the combination of several CNN models. However, the computational power and time required for training and testing should be considered for practical applications. In order to be able to use deep learning methods for real-time applications such as polyp segmentation tasks by embedding models into specific devices, e.g., a medical capsule robot, attention needs to be paid to the development of an efficient overall model during the implementation phase. An efficient model should have relatively low requirements for hardware, computational power, and training time. CNNs such as DeepLab_v3 are computationally intensive, as they involve numerous learnable parameters [13]. From 2014 onwards, benchmarking for ImageNet performance reached a bottleneck, where the slight increases in performance of a network were due to its deeper and more sophisticated architecture [14].

The goal of our study is to implement a less complex network with a minimal trade-off on the segmentation results such as accuracy, sensitivity, and specificity. However, with the existing computer hardware technologies, embedding a simple (e.g., AlexNet and Visual Geometry Group (VGG)) or a high accuracy and sensitivity model (e.g., ensemble models) into a real-time application device for segmentation tasks is nearly impossible. That is because the fully connected layer contributes the largest number of trainable parameters to the network architecture. We therefore used a SegNet Visual Geometry Group-19 (VGG-19) [15] as the framework for our proposed automatic polyp segmentation model in this research work as it is well known for its smaller number of network parameters and the shorter times needed for network training. In order to reduce the network training time, SegNet uses a unique and efficient method of retaining pixel information from the downsampling path to use in the upsampling path.

Although SegNet has been popular due to its simple and efficient network architecture, its main drawback is its lower performance than other, more sophisticated, and deeper networks. SegNet is a more linear architecture than other networks such as U-Net and fully convolutional networks (FCNs), which makes it less promising in segmentation performance due to missing pixel information. Hence, in this study, we modify the SegNet architecture to enhance the segmentation performance in terms of accuracy, sensitivity, and specificity.

In summary, the main contributions of this paper are as follows:

1. We propose a novel CNN based on SegNet VGG-19 encoder–decoder architecture for automatic polyp segmentation. The goal of this work is to improve segmentation performance such as accuracy and sensitivity in the original SegNet model and reduce the model’s complexity without losing too much performance when compared to other existing methods. We introduce three modifications, i.e., skip connections, 5 × 5 convolutional filters, and concatenation of four dilated convolutions applied in parallel form on SegNet VGG-19.
2. We train and evaluate our proposed model on a combination of three publicly available datasets, i.e., CVC-ClinicDB, CVC-ColonDB, and ETIS-Larib Polyp DB. Our proposed model manages to outperform the original SegNet VGG-19.
3. Our proposed model performs as well as or better than previous schemes in the literature. Our segmentation performance achieved accuracy, sensitivity, specificity, precision, mean intersection over union, and dice coefficient of 96.06%, 94.55%, 97.56%, 97.48%, 92.3%, and 95.99%, respectively. Although our proposed model slightly underperforms compared to one of the methods in the literature, our network has less complexity, that is, our method has almost half of the trainable parameters as that method.

The rest of the paper is organised as follows. Section 2 discusses previous works related to polyp segmentation. Section 3 reviews the SegNet model used in this research. The limitations of this model are explained and elaborated on in this section, and the modifications made to the existing SegNet to tackle these limitations are explained. In Section 4, our segmentation results and the performance of the modified SegNet are evaluated and analysed in terms of the network training time and evaluation metrics such as accuracy, sensitivity, and specificity. Finally, Section 5 summarises and concludes the paper.

## 2. Related Works

The conventional methods proposed in earlier studies have often extracted intrinsic polyp features determined by the researcher, typically consisting of texture information, colour, edges, and contours. The authors in [16] used principal component pursuit (PCP) to fragment images into low rank and sparse forms for background subtraction. In the last stage, the image was segmented via an active contour applied to the background-subtracted image. Another active contour segmentation method was proposed in [17], in which the active contour framework was modified to work without edges. The authors claimed that this remodelled active contour framework had the advantages of the contours merging and splitting on their own; on the contrary, the old snake model can merely extend and straighten without much versatility. Polyp segmentation based on contour region analysis was proposed in [18]. The authors extracted features using the curvature characteristics and the colours of the polyps, the shape of their edges, and their areas. Edges identified as polyps would further detect their start and end points linked to create the polyp area. Traditional methods have had limited success due to the need for researcher expertise and the numerous factors affecting the environment of the colon; in particular, there are difficulties in differentiating between polyps and non-polyp backgrounds, which have very similar characteristics.

Deep learning techniques have been widely adopted in the image analysis domain and have outperformed traditional methods. The features of polyps can be extracted from training datasets by fitting a non-linear neuron-crafted network with non-convex objectives. The selected features can be further simplified by the intrinsic deep architecture of a neural network for classification at a later stage. The significant achievements of CNNs have led to an increase in popularity, and these models have become prevalent in medical image analysis. The authors of [19] developed and compared FCNs based on three different network architectures (FCN-AlexNet, FCN-GoogLeNet, and FCN-VGG). These CNNs were converted into FCNs through the replacement of fully connected and classification scoring layers by 1 × 1 convolution, binary scoring, deconvolution, and dense prediction layers. In [20], traditional handcrafted methods were combined with deep learning approaches for polyp segmentation. FCNs were used for the extraction of polyp features and the generation of region proposals, which were then refined using texton patch representation to separate polyp and non-polyp regions before classification with a random forest algorithm. In [21], the identification of polyp features and the spatial resolution recovery of polyp images were realised using FCN-8, a method that upsamples the feature maps generated from the last two pooling layers and the last convolution layer. These upsampled feature maps containing polyp regions were then post-processed with Otsu thresholding to give the largest connected component, which was used for polyp segmentation. The authors of [22] proposed an FCN for polyp segmentation inspired by U-Net and FCN. Convolution, Rectified Linear Unit (ReLU), and pooling layers were built for feature extraction, and a fully connected convolutional layer was added at the end of the feature extraction stage to increase the comprehensive connectivity between the pixels of the image. In the prediction map reconstruction phase, the feature maps were upsampled to the original input image resolution via deconvolution. A skip connection structure was used in [22] to concatenate the low-level fine features from the downsampling path into high-level coarse features in the upsampling path. The FCN architecture has a drawback in that spatial information on the polyp image is lost in the pooling layers. When the global context is lacking, an FCN typically captures and generates prediction maps based on features at a high abstraction level at the centre of the object of interest, resulting in a rough and discontinuous segmentation effect [23].

In [24], a fully convolutional densely connected convolutional networks (DenseNet) approach was proposed for polyp segmentation. The proposed network consisted of an FCN, a skip connection, a traditional dense connection, and pruned dense connection in dense blocks. However, due to the dense network architecture, which generates more feature layers, this approach required a relatively large amount of memory to store the feature layers. The authors of [25] used a LinkNet with a new combination of colour spaces as the network image input. The red and green components were chosen from the red, green, and blue (RGB) colour space, and were combined with b* from the International Commission on Illumination (CIE)-L*a*b* colour space to give a new colour space for the input image. The prediction maps output from LinkNet were post-processed using the convex hull algorithm. Lastly, the largest connected component was selected for polyp segmentation. A Mask Regional-CNN (Mask R-CNN) was implemented with a residual neural network (ResNet)-101 backbone for feature extraction in [26]. A transfer learning method was implemented to train the Mask R-CNN, which was previously pre-trained with the Microsoft Common Objects in Context (MS-COCO) image dataset and colorectal image datasets for polyp segmentation. The authors of [27] modified two deep neural networks: DeepLab_v3 with long short-term memory (LSTM), and Segnet with LSTM. LSTMs can retain localisation information and preserve information, thus preventing it from vanishing in the encoder networks. An LSTM has memory cells and three different gates: an input gate, a forget gate, and an output gate. The input gate determines which new information should be written into memory cells, while the forget gate determines which memory cell should be overwritten, and the output gate determines which information should be released. The method proposed in [27] did not obtain the expected level of performance; SegNet + LSTM obtained 77.28% in mean intersection over union (IoU), performed worse than DeepLab_v3 + LSTM which obtained 79.30% in mean IoU, and the latter method could not connect the interrelationship of features between ResNet, atrous convolution, and atrous spatial pyramid pooling. The authors of [28] proposed two methods, i.e., Enhanced FCN-8 (EFCN-8) and Enhanced SegNet (ESegNet). The ESegNet obtained mean intersection over union (mIoU) and accuracy of 72.8% and 93.5%. The EFCN-8 obtained 76.7% and 94.9% on mIoU and accuracy, respectively.

The authors of [29] proposed a loss function called the eigenloss by combining seven loss functions: Jaccard, Dice, binary cross entropy, binary focal, Tversky, focal Tversky and Lovász hinge. These losses were combined in a linear way by extracting the coefficients using principal component analysis (PCA). The eigenloss was tested on two networks (U-Net and LinkNet) and two backbones (VGG-16 and Densenet121), and it was found that the LinkNet–Densenet121 yielded the best result. This approach obtained accuracy, sensitivity, and specificity of 94.59%, 80.32%, and 99.05%, respectively. However, the sensitivity in this approach is not promising enough. Sensitivity is important in polyp segmentation as it represents the ability to correctly identify the polyp region.

In [30], the authors proposed a Mask R-CNN as a method of polyp segmentation. They used Res2Net, a modified version of ResNet, as the bottleneck structure for the Mask R-CNN. In Res2Net, the 3 × 3 filters found in the bottleneck block of the old ResNet were replaced with smaller filter groups connected in a hierarchical residual-like manner. Mask R-CNN is based on Faster R-CNN and a feature pyramid network (FPN), which can be regarded as a two-stage process for segmentation. The authors of [31] proposed an ensemble model consisting of two Mask R-CNNs with a Resnet50 (25.6 million trainable parameters) and a Resnet101 (44.6 million trainable parameters) as backbone architectures. A bitwise combination was used to combine the two networks into an ensemble model with the aim of improving the segmentation performance. The authors of [32] proposed an ensemble of three deep encoder–decoder networks with DeepLabV3X, in which several different convolution strides are used in each component network to achieve finer feature extraction for medical object segmentation tasks. Each of these single deep encoder–decoder networks was trained on input datasets with different resolutions. The resulting ensemble model was able to extract the important features from a polyp. There are both advantages and disadvantages of using the ensemble method, and these depend on the objectives of the research. It has the advantage of being able to combine several networks to distinguish more detailed information. A combination of networks can complement each other and offset the other’s disadvantages. However, models combining several different networks, such as the Resnet50 and Resnet101 with Mask R-CNNs in [31] that have approximately 70.2 million trainable parameters, and the three continuous multiple deep encoder–decoder networks in [32] that have approximately 137.4 million trainable parameters, are extremely complex. The time required for the training and image processing on a complex ensemble model will be longer than for a model consisting of a single network.

The authors of [33] created an ensemble of three CNNs, the U-Net-VGG, SegNet-VGG, and Pyramid Scene Parsing Network (PSPNet), as a polyp segmentation model. A weight voting method was used to combine the segmentation results from these three networks. The weight distributions among the U-Net-VGG, PSPNet, and SegNet-VGG that provided the best results in [33] were 2:2:1 and 3:3:2 on the cvc300 dataset, 3:1:1 on CVC-ClinicDB, and 3:2:2 on ETIS-LaribPolypDB. The U-Net and SegNet were estimated as having 27.5 million and 29.5 million trainable parameters, respectively [28], whereas the PSPNet with ResNet-50 as the feature extractor was estimated to have 44.6 million trainable parameters [4]. The ensemble model in [33] therefore has a total of approximately 101.6 million trainable parameters. The approach in [33] performs well, as the use of three different networks in one model enables it to learn different polyp features and to aggregate multi-scale contextual information from the same database. The disadvantage of the ensemble methodology in [33] was that the three different networks, U-Net, SegNet, and PSPNet, needed to be trained separately. This approach therefore requires a great deal of effort and computational power.

## 3. Materials and Methods

In this section, the datasets and method used in this study are discussed. Section 3.1 discusses the datasets used to train and test our proposed method in detail. Section 3.2 introduces SegNet VGG-19, the framework architecture for our proposed model. Section 3.3 explains the modifications that were made to SegNet VGG-19 as the final polyp segmentation model. The drawbacks of the original SegNet VGG-19 and the ways in which our improvements can resolve or at least minimise these problems are discussed in Section 3.3.

### 3.1. Datasets

The proposed method was evaluated on three publicly available datasets: CVC-ClinicDB [34], CVC-ColonDB [35], and ETIS-Larib Polyp DB [36]. CVC-ClinicDB consists of 612 colonoscopy images with a resolution of 384 × 288 extracted from 29 different video sequences. CVC-ColonDB contains 379 colonoscopy images with resolution 574 × 500, generated from 15 different video sequences, while ETIS-LaribPolypDB contains 196 colonoscopy images with resolution 1225 × 966, generated from 34 different video sequences. Each video sequence represented a subject (polyp) and background. These datasets are summarised in Table 1.

**Figure A1:**
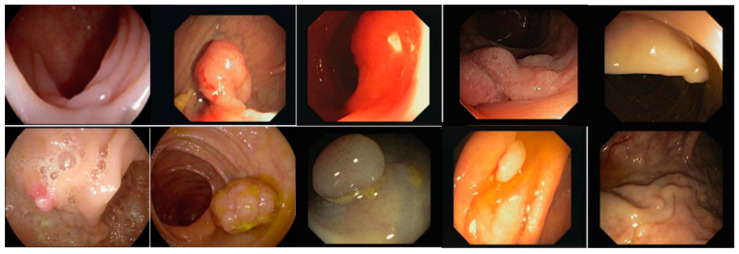
Samples of colonoscopy images with a variety of polyp conditions.

All of the colonoscopy images were associated with manually annotated polyp masks drawn by experts, and each image contained at least one polyp that was associated with its own individual polyp masks. A total of 1187 images were collected, and 70% was randomly selected to train the segmentation model network, while the rest was used to test the model. The breakdown for training and testing images on the three datasets is depicted in Table 1. These three databases contain a variety of images of polyps in terms of their condition, the light intensity, the angle of view, and the size and structure. Using these images means that our model will be more diversified and can tackle polyp segmentation tasks in a range of environments. Examples of colonoscopy images showing the diversity of polyp conditions can be found in Figure A1 in Appendix A.

### 3.2. Basic SegNet Architecture

SegNet is a well-known architecture for semantic segmentation computer vision tasks. Unlike very deep networks such as DeepLab_v3, SegNet requires a shorter training time due to its smaller number of trainable parameters. SegNet architecture is normally built up with VGG and its inverse as encoder and decoder networks [13]. Most prior SegNet research has used VGG-16 as the model baseline; however, a SegNet VGG-16 is unable to extract sufficient useful details from an image to differentiate between a complex polyp structure and a visually similar background consisting of the colon lining. One way to counter this limitation is to use a deeper CNN structure to increase the network complexity, which will allow the network to encode more image information, leading to better general polyp segmentation performance. Thus, in our method, a SegNet architecture model based on the VGG-19 framework [37] is modified for the task of polyp segmentation.

The SegNet architecture consists of a VGG-19 encoder for the downsampling path and its reverse for the upsampling path. The encoder network of a SegNet VGG-19 contains a series of convolution blocks constructed from convolutional layers, batch normalisation, and ReLU activation layers. Consecutive convolution operations are applied to the input colonoscopy images to extract hierarchical polyp features from low-level to high-level local information. The convolutional layers are responsible for detecting and extracting local features at all of the locations in the input images through filters. Convolutional filters convolve around input images to extract features with increasing relevance from texture to higher-order task-related features. The convolution operation and bias inclusion can be expressed as:

(1) $$Y_{i,j}=\sum_{C}\sum_{m,n}k_{m,n}X_{C,i+m,j+n}+b,$$

where *Y* is a feature map. A set of convolutional filters *k* is convolved with the input image *X*, and the biases *b* are included after the convolution operation. The terms *i* and *j* represent the *i*th row and *j*th column element of the image, while *m* and *n* represent the *m*th row and *n*th column element of the convolutional filter. The number of convolutional filter channels is denoted as *C*. In this way, the input dimensions and numbers of parameters can be reduced simultaneously, thus making the network equivariant with respect to input translations.

The batch normalisation layer that is applied after the convolutional layer makes the network more generalised [38] by executing a kind of lateral inhibition across all output feature maps from the prior convolutional layer. A network may encounter the issue of internal covariate shift, in which there is a change in the distribution of the inputs to the activation layers due to a change in parameters as the network parameters are frequently updated during training. When there is a difference in the input distribution between the mini-batches used for training, the network layers need to adapt to this new distribution, meaning that the network requires more time to converge. Batch normalisation is included to coordinate the weight updates and to standardise the image input to the network layer per mini-batch by rescaling the pixel’s activations. Two new trainable parameters, the mean and standard deviation, are introduced to normalise the activations of the output feature maps in mini-batch form from the previous layer to a zero mean and unit variance. These two parameters enable the network to scale and shift the standardised mini-batch output.

The ReLU, which is applied after batch normalisation, is responsible for extracting the hierarchy of the polyp features from the normalised output feature maps from the convolutional layer. This layer limits or cuts off features that are not useful and retains the remainder to speed up the network training process. A *ReLU* has a simple definition [39], as follows:

(2) $$ReLU\left(Y\right)=\max\left(0,Y\right),$$

where *Y* is the feature map output from the previous batch-normalised convolutional layers. Max pooling is performed after each convolution block on the encoder path. The subsequent convolution block can extract more contextual and spatial invariant features with higher complexity from the training images. The max pooling layer returns the maximum value from the portion of the image covered by the kernel. It reduces the spatial resolution of the convolved features, thus helping the network to decrease the number of computations and the training time. After the input image has been passed through the convolution block and max pooling layers (also known as the encoder network) and has been downsampled, high-level polyp features can be obtained.

SegNet has a decoder network that corresponds to the encoder network. This decoder network upsamples input feature maps from the max pooling layers in the encoder network. The indices and sizes from max pooling in the downsampling paths are passed to max unpooling in the upsampling paths. Deconvolution, a parametric method of recovering the resolution of the images, is performed after max unpooling. In this way, the activation maps representing the finer polyp features extracted from the encoder can be reconstructed as a binary segmentation map with a similar resolution to that of the input image. This unique scheme in SegNet can efficiently upsample feature maps in its decoder network, whereas other network architectures such as DeepLab_v3 and U-Net use transposed convolution as their upsampling method. Transposed convolution is a type of learnable upsampling method that requires a longer training time. It essentially operates in a similar way to a convolutional layer, although it inserts zeros between each row and column of the input maps for enlargement.

CNN-based algorithms are trained via pixel-to-pixel matching between each obtained probability map and the corresponding ground truth. At the end of the SegNet model, a binary cross-entropy loss function is applied, which calculates the differences in the probability distributions of the polyp and non-polyp pixels. In order to create an accurate segmentation network, a smaller cross-entropy is desired. The results from SegNet are not necessarily in the form of a probability distribution, and Softmax regression is therefore used to change the output into this form. Softmax is an activation layer that is used to produce a discrete probability distributor vector, and can be conveniently combined with the cross-entropy loss.

### 3.3. Modifications to the SegNet Architecture

**Figure 1:**
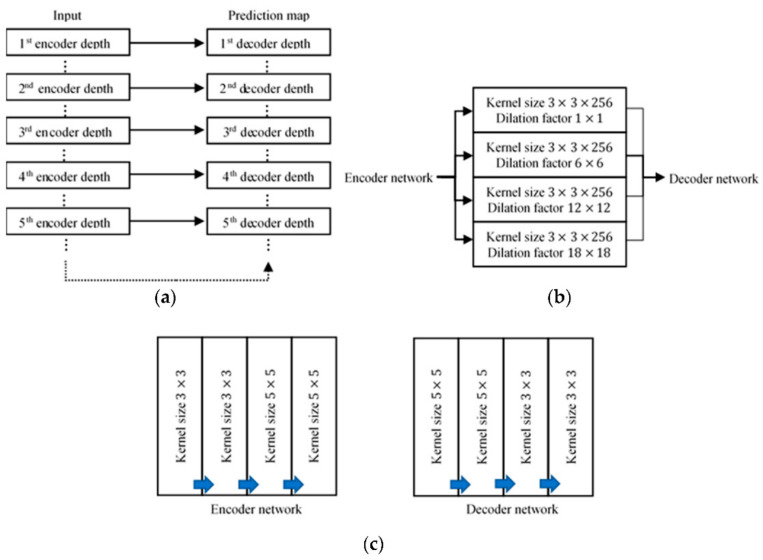
Modifications made to the original SegNet Visual Geometry Group-19 (VGG-19) structure: (**a**) overview of skip connections (solid arrow) introduced in our modified SegNet; (**b**) parallel dilated convolutions used at the end of the model encoder network; (**c**) the 5 × 5 kernel size convolution blocks used in our modified SegNet.

In our modified SegNet scheme, we changed the original SegNet VGG-19 structure by adding skip connections and 5 × 5 convolutional filters, and concatenating the parallel dilated convolutions. These changes were carried out with the aim of overcoming the limitations of SegNet in terms of detecting finer features for the two classes, polyps and non-polyp backgrounds. Modern CNNs apply successive convolutional layers and pooling layers in order to aggregate multi-scale contextual information until a global prediction can be made. However, a weakness of the original SegNet is that the subsampling and max pooling layers tend to induce losses of the potential useful spatial features and reduce the gradient along the downsampling path, due to its more linear architecture [29]. If the output feature maps of the encoder were directly upsampled, the outcome prediction map would be very coarse and noisy, due to the lack of a global view. A local view is used to detect the location of the object of interest, while a global view detects the identity of that object. The original SegNet model is effective in terms of the local view, but not the global view. To tackle this issue, skip connections were introduced into our proposed SegNet to connect the downsampling paths to the upsampling paths, with the aim of preserving information on the polyp locations. Our proposed model combines the outputs from all the five encoder depths with the inputs of their corresponding decoder depths and enables feature maps to be fed and reused with no modifications. This enabled the upsampling paths to recover fine-grained information from the downsampling layers and to compensate for the pixel information losses caused by the max pooling layers. In this way, the decoder network is able to leverage stronger relevant encoder feature maps, thus allowing the proposed network to capture both global and local polyp information for better boundary segmentation without increasing the number of network parameters or adding relearning operations. Since skip connections are directed to the decoder network through convolution operations from the encoder, they help to speed up the network training process and improve the gradient flow through the effective backpropagation of error corrections to the encoder network to minimise the gradient diminishing issue, since skip connections are directed to the decoder network from the previous encoder convolution operations. The inclusion of skip connections in the SegNet model is shown in Figure 1a.

The original SegNet includes 3 × 3 small kernels in the convolution layers. Although a 3 × 3 filter can extract a great deal of local information and features from shallow layers, noise will also be captured, which is less useful. Directly feeding these feature maps into deeper layers can reduce the performance of polyp segmentation due to confusion with non-polyp background information, in this case noise. Our proposed network was modified by adding two convolution blocks with a 5 × 5 kernel size to both the first two encoder depths and the last two decoder depths, to retain the symmetrical network structure of SegNet. These 5 × 5 convolution blocks were added after the initial 3 × 3 convolution blocks on the encoder side, and before the initial 3 × 3 convolution blocks on the decoder side. The aims of this modification were to create more stable features and to filter out some of the complex background noise. Increasing the number of convolutional layers makes the network more non-linear, thus more discriminative functions are used to extract the polyp features. This modification was made specifically at the input of the encoder network and the corresponding location in the decoder network of our proposed model, as most of the decisive local information on the polyp features is detected in the shallow layers. In this way, the more valuable features that are extracted can be used in the deeper network for more effective pixel classification. The implementation of this modification is shown in Figure 1c.

**Figure A2:**
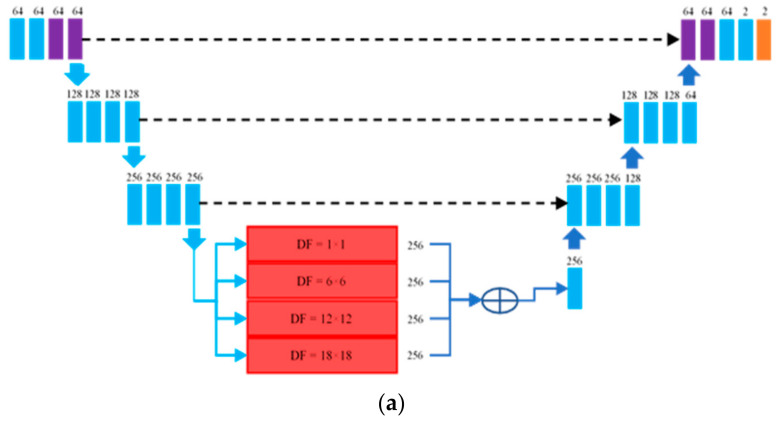

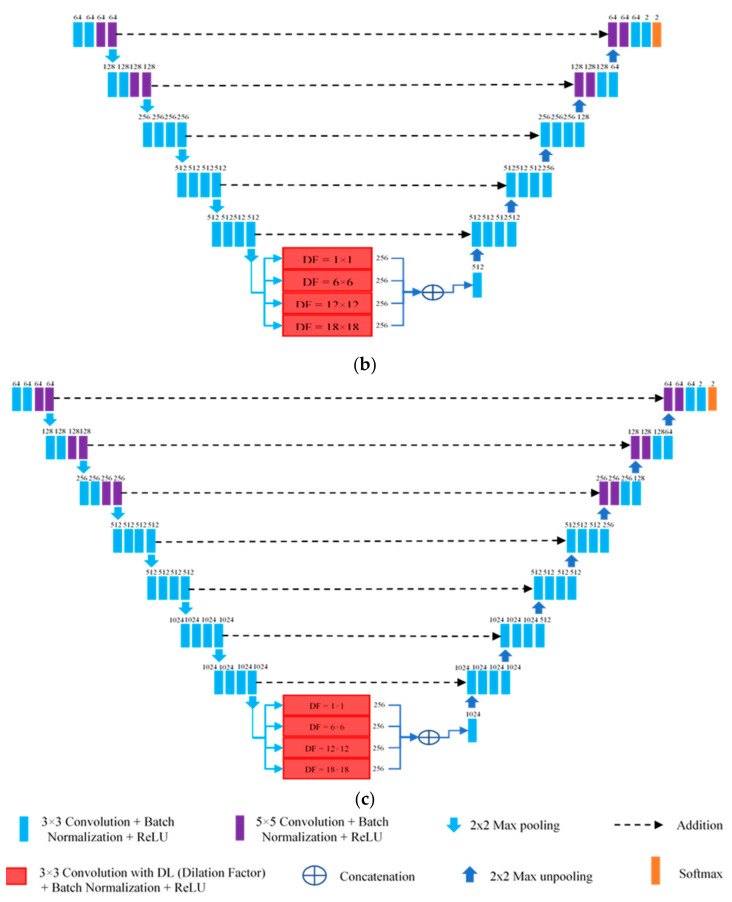
An overview of our three proposed networks: (**a**) 100-layer network; (**b**) modified SegNet (154-layer network); (**c**) 208-layer network. The small numbers represent the number of image channels used in each convolution block.

In our modified SegNet model, wider networks were created at the end of the encoder path by concatenating four dilated convolutions in different dilation factors (1, 6, 12, and 18) applied in parallel form. The filter weights were expanded and aligned according to those specified dilation factors. The placement of the weights in the convolutional filter can be adjusted within specific intervals to alter the filter size, thus enlarging the receptive field without losing the resolution. The dilated convolution operation [40] can be written as:

(3) $$Y=\left(X{*}_{l}k\right)\left(p\right),$$

where *Y* is the output feature map, *X* represents the input image, *k* represents the number of convolutional filters, *p* represents padding, and *l* represents the dilation factor. These dilated convolutions are applied in a parallel form and the outputs are then concatenated. The concatenated output feature maps are then passed to a convolution block to create a suitable dimension for the feature maps that will later be unpooled in the decoder network. As mentioned in [41], the resolution of the receptive field is critical in order to resolve small objects. Even with the help of skip connections, feature information on small objects is hard to recover, and it is difficult to identify polyps, as they may have all kinds of shapes and sizes. Dilated convolutions applied in parallel form were included in our modified SegNet to enlarge the receptive field of the network in order to abstract contextual information and fine details by processing input maps with various resolutions. By concatenating these together, spatial information can be aggressively consolidated across the inputs while retaining the same resolution. This architecture can discover and learn new fine-grained feature details to recover lost vital polyp information, and particularly in relation to edges and boundaries. Figure 1b shows the parallel dilated convolution architecture used in our model. An overview of the whole modified SegNet VGG-19 is given in Figure A2 in Appendix B.

## 4. Results and Discussion

### 4.1. Network Training Protocol

We propose a model based on a modified SegNet, with VGG-19 as the framework architecture. Our model was trained in a supervised manner using ground truths that were manually annotated and verified by expert clinicians as a reference. Of 1187 images, 831 were randomly selected for training, i.e., 437 (CVC-ClinicDB), 260 (CVC-ColonDB), 134 (ETIS-Larib Polyp DB), and the remaining 356 were used for testing, i.e., 175 (CVC-ClinicDB), 119 (CVC-ColonDB), 62 (ETIS-Larib Polyp DB) to evaluate our model. There were no intersections between the testing and training sets. We trained our networks using stochastic gradient descent to update the network weights. All of the models were trained with the following parameter settings: momentum 0.9, learning rate 0.001, 100 epochs, and a mini-batch size of four. The learning rate was reduced by a factor of 0.3 after every 10 epochs. Our models were implemented and trained in Matlab with an Nvidia GeForce RTX 2080 Ti GPU.

### 4.2. Network Performance Evaluation

A network was formulated to produce dense pixel-wise polyp segmentations on testing images for performance evaluation. In the task of polyp segmentation, there are two class labels, polyp and non-polyp. By comparing the segmentation results with the reference label map, four measurements can be obtained: true positive (TP), false positive (FP), true negative (TN), and false negative (FN). TP represents polyp pixels that are predicted as polyps, while FP represents non-polyp pixels that are predicted as polyps, TN represents non-polyp pixels that are predicted as non-polyps, and FN represents polyp pixels that are predicted as non-polyps.

We evaluated and quantified the effectiveness of our models in terms of the accuracy, sensitivity, specificity, precision, mean IoU, dice coefficient, and F2 score. Accuracy means the proportions of true positives and true negatives over all the datasets in the test, which corresponds to the correct prediction of the polyp and non-polyp pixels. The higher the accuracy, the better the segmentation method. The sensitivity (also known as recall) is the proportion of positive values returned by the segmentation compared to the positive values based on the ground truths. The specificity represents the proportion of negative values produced by the segmentation method compared to the real negatives, as determined by the ground truths, while the precision measures the ratio of the number of true positive values to the number of positive values predicted by the segmentation method. The mean IoU reflects the similarity between the ground truth and the predicted results from the method. The dice coefficient indicates how well the predicted boundary of each class aligns with the true boundary, and the F2 score measures the accuracy of a test based on the precision and recall. The formulae for all the evaluation metrics used here can be found in Appendix D.

### 4.3. Analysis of Training and Testing Results

In addition to our modified SegNet models (154 layers), we also compared two further modified SegNet models with different numbers of layers (100 and 208 layers). The aim of this comparison was to determine the best-fitting number of network layers and depths with different dimensions of image channels for our modified SegNet in the task of polyp segmentation. All three modified SegNet models had a similar network structure, and the only difference lies in the depth of the encoder and the corresponding depth of the decoder. An overview of all the three networks is shown in Figure A2 in Appendix B.

**Figure 2:**
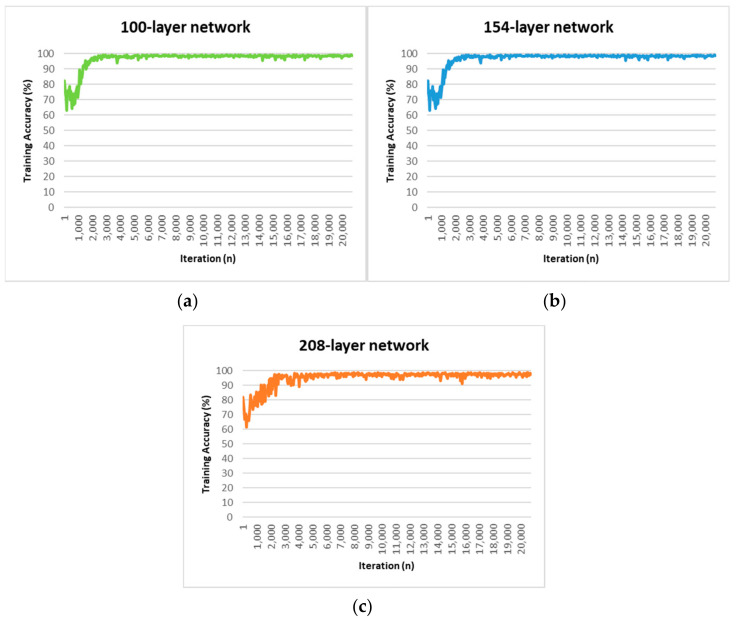
Training accuracy curves for the three networks: (**a**) 100-layer network; (**b**) 154-layer network; (**c**) 208-layer network.

The 100-layer network consisted of three-layer encoder depths, made up of 64, 128, and 256 image channels. This network was implemented based on our modified SegNet, except that the four- and five-layer encoder depths were eliminated, together with their corresponding decoder networks. This elimination was performed to reduce the complexity of proposed network. The four-layer and five-layer encoder depths of our modified SegNet have 512 image channels which increase the network’s complexity. The 154-layer network had five-layer encoder depths, made up of 64, 128, 256, and two 512 image channels. The 208-layer network was an extended version of our modified SegNet, and consisted of seven-layer encoder depths, with 64, 128, 256, two 512, and two 1024 image channels. The decoder paths in all three networks were symmetrical with respect to the encoder paths. We compared the segmentation results for the three networks and the time required to train each network. All three networks were fully trained for 100 epochs (20,775 iterations) and stabilised at a training accuracy of around 96–98%. The curves for the training accuracy for all three networks are shown in Figure 2, and the training times for each network are given in Table 2.

For the same training dataset and the same number of training epochs, the 208-layer network required the longest time for training (475.19 min), followed by the 154-layer network (355.52 min), and finally the 100-layer network (331.16 min). Deeper networks required longer training times, as there were more parameters to be trained. The results for the evaluation metrics for the models with different numbers of layers are given in Table 3.

Based on the results shown in Table 3, the 154-layer network gave the best overall performance. We can observe that, on average, this network obtained an accuracy of 96.06%, a sensitivity of 94.55%, a specificity of 97.56%, a precision of 97.48%, a mean IoU of 92.3%, a dice coefficient of 95.99%, and an F2 score of 95.12%. Although the 100-layer network achieved specificity and precision values that were 0.2–0.3% higher, the 154-layer network outperformed it by a large margin on the other evaluation metrics. The 208-layer network performed worst on each evaluation metric. The values for the training and testing accuracy obtained by the 100-layer network were 98.49% and 93.84%, respectively, showing a difference of 4.65%. The 154-layer network yielded values for the training and testing accuracy of 98.49% and 96.06%, with a difference of 2.43%. The 208-layer network gave values of 97.59%, and 92.09%, with a difference of 5.5%. The segmentation accuracies for all three networks on the testing dataset are shown in Table 4.

The training accuracy curve for the 100-layer network is similar to that of the 154-layer network, although poorer performance is seen on the testing set. Table 4 shows that 70 of the 356 images from the testing dataset resulted in an accuracy of below 90% for the 100-layer network. For the 154-layer network, 52 of the 356 testing images were below 90% in accuracy, 18 fewer than the 100-layer network. We speculate that the 100-layer network had an insufficient number of trainable parameters to fully learn and recognise the more detailed polyp features, although it performed similarly to the 154-layer network in the training stage.

The 100-layer network has 10.4 million trainable parameters, almost five times fewer than the 154-layer network. The 100-layer network was not sufficiently complex or deep to extract the finer polyp information from new unseen images, and therefore performed poorly on the testing dataset. As the network becomes deeper, we see an increase in the number of trainable parameters, which generally improves the performance of the model, but this is not always true for the 208-layer network; surprisingly, this one performed the worst of all three networks, possibly due to overfitting. Overfitting issues mainly occur in a very deep networks due to their extreme complexity, as they have a tremendous number of trainable parameters that may be biased towards a specific dataset or batch of images. The 208-layer network has 206.7 million trainable parameters, almost four times and 20 times more than the modified SegNet and the 100-layer network, respectively.

Overfitting can be observed for the 208-layer network from the training accuracy curve in Figure 2. The training accuracy for both the 100-layer and the 154-layer networks increased smoothly until convergence was reached, while for the 208-layer network, this fluctuated throughout the training process. The reason for this could be that the network is biased towards a certain batch of training images, where most of the trainable parameters were trained to suit the features of polyp extracted on that particular batch of images. In this case, when the next batch of training images is fed into the network to update the trainable parameters, the network cannot find a global minimum for the trainable parameters that is suitable for the entire training dataset. Overfitting can also be seen in a network when the testing performance is far poorer than the training performance, i.e., the segmentation results on the testing images are poorer than expected. The network has a training accuracy of 97.59% and a testing accuracy of 92.09%. We can observe from Table 4 that the 208-layer network had an accuracy of below 90% on 84 of the 356 testing images (14 and 32 more than for the 100-layer and 154-layer networks, respectively). Overall, the 154-layer model outperformed the 100-layer and 208-layer networks by an average of 2.7% and 5%, respectively.

### 4.4. Discussion of Results and Comparison with Previous SegNet Research

Based on the results of this comparison, the 154-layer network was used in the subsequent experiments as our modified SegNet model. An original SegNet with a VGG-19 architecture was also implemented for comparison with our modified SegNet. In order to achieve a fair comparison, all of the datasets and training parameters were identical for both the original and the modified SegNet models. We also compared the segmentation performance of our model with the networks in [27,28], which used SegNet as their basis. A comparison of the results is given in Table 5, and it can be seen that our modified SegNet model gives a clear improvement over other methods.

**Figure 3:**
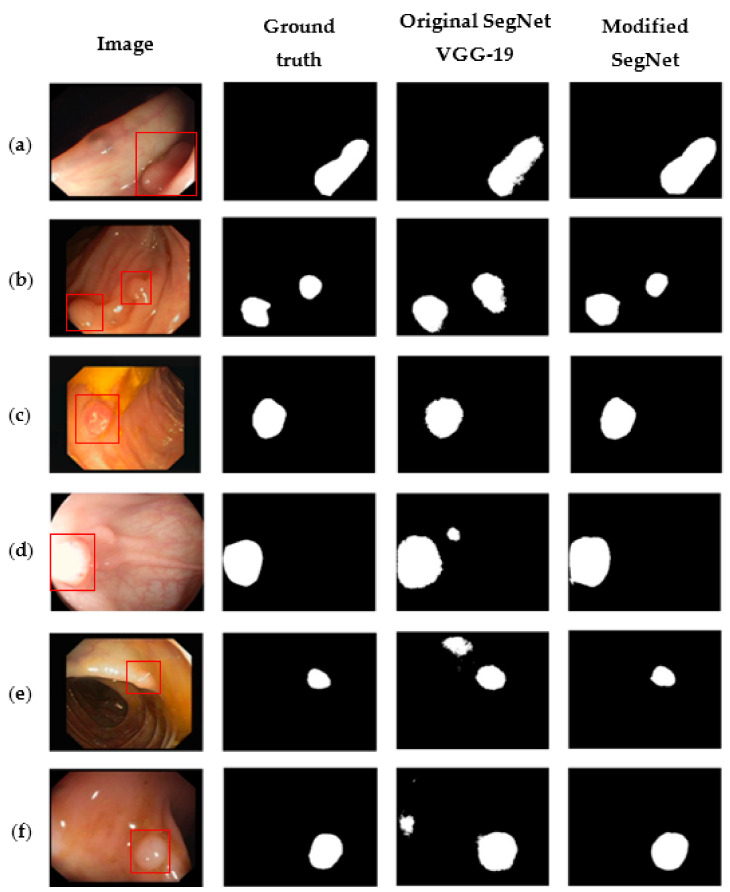
Comparative examples of polyp segmentation results. The first column contains raw images, the second column contains the reference ground truths, the third column shows the segmentation results from the original SegNet VGG-19, and the final column shows the segmentation results from our modified SegNet. The red boxes in the raw images (**a**–**f**) indicate the location of polyps. The white patches in the second, third, and final columns indicate the segmented polyps, the black areas indicate the non-polyp regions.

It can be seen from Table 5 that our proposed model performed better than the other SegNet models, and also better than the models proposed in [27,28]. This improvement may be due to the use of VGG-19 as a network backbone, whereas VGG-16 was used in [27,28]. There are more convolutional layers in VGG-19 than VGG-16, and these additional layers may have enabled our model to learn more of the features of polyps. The score of 73.72% for the IoU indicates that our model obtained more true positive polyps, whereas the scores for the other methods were well below 70%. In terms of the mean IoU, only our modified SegNet model managed to exceed 90%. Figure 3 compares the results from our modified SegNet to those of the original SegNet VGG-19. The red boxes in the first column in Figure 3 indicate the location of polyps in the raw images.

As shown in rows (a) to (c) of Figure 3, the experimental results from our method are smoother at the edges of the polyp and less over-segmented as compared to the original SegNet model. This phenomenon may be due to the use of skip connections in our improved model. The fine-grained information from the encoder network can compensate the vanishing pixel information along the network, thus giving a smoother polyp segmentation effect. We can also observe from rows (d) to (f) in Figure 3 that our modified SegNet model gave fewer false positive regions compared to the original SegNet model. Some background regions were incorrectly recognised as polyp regions by the original SegNet, according to the ground truth, whereas our modified SegNet managed to correctly identify all the locations and the numbers of polyps in the same images. We believe the reasons for this improvement were our modifications to SegNet, and particularly the use of convolutional layers with a kernel size of 5 × 5 and the parallel dilated convolutions in the final encoding layers. We speculate that the incorporation of these convolutional layers in the two shallower convolution blocks in both encoder and decoder networks help to further remove noise and stabilise the prominent features of polyps. The use of dilated convolutions applied in parallel form enlarges the receptive field of the model and enables it to extract and aggregate multi-scale contextual information. In this way, more spatial information on the polyp features can be consolidated, resulting in better segmentation outcomes in terms of discriminating between polyp and non-polyp pixels.

We visualise and compare the activations of SegNet VGG-16, SegNet VGG-19 and our modified SegNet model. Activations of different SegNet layers can be displayed and visualised when a colonoscopy image is fed to the model. Through visualisation, the features learned by the network can be discovered by observing and comparing the activation areas with the original image. The visualisations were compared separately for the encoder network, the intermediate sector between the encoder and decoder, the decoder network, and the final prediction map. Our modified SegNet model is more capable of capturing more stable and higher continuity features for the polyp edges as a whole than SegNet VGG16 and SegNet VGG-19. The activation visualisation shows that the strongest white pixel activations were accurately determined to be within the polyp area. In the visualisation of intermediate sector between the encoder and decoder, our modified SegNet model gave different receptive field of output activation maps. The concatenation of four dilated convolution blocks gave the strongest pixel activation within the correct location of polyps. In the visualisation of the decoder network, our modified SegNet model also performed better than SegNet VGG-16 and SegNet VGG-19. The activation maps of our modified SegNet model contained less noise, and were more accurate and more centralised on the polyp region. For more information about activation visualisation, the reader can refer to Appendix C.

### 4.5. Discussion of Results and Comparison with Other Previous Research Works

In addition to the networks in [27,28], we also compared our modified SegNet model with other recent CNN-based methods. A comparison of the performance of all the networks in given in Table 6.

The comparison above shows that our method outperformed most other methods. Our approach performed better than the model in [31] for every performance index, with a significantly higher value for the sensitivity (by 18.3%), and a better precision and mean IoU, with gains of 0.25 and 0.33, respectively. Compared to the model in [32], although our method had a lower accuracy and sensitivity (by 2.64% and 1.65%, respectively), it yielded better results for the specificity, precision, and dice coefficient, with improvements of 3.36%, 2.48%, and 6.89%, respectively. Our method also outperformed the two most recent methods identified in our literature review, i.e., those in [29] and [30]. As we can observe from Table 6, although the model in [29] yielded a specificity that was 1.49% higher than our method, our model outperformed it overall by a significant margin, with values of 96.06% for accuracy, 94.55% for sensitivity, 97.48% for precision, 92.3% for the mean IoU, and 95.99% for the dice coefficient, compared to values of 94.59%, 80.23%, 88.65%, 71.84%, and 79.07%, respectively. Based on a comparison with [29] in terms of the performance metrics, we can see that our method detected more TP regions and segmented fewer FP areas. For the task of polyp segmentation, it is essential to improve the TP segmentation rate, as the sensitivity is the factor that determines the performance of the model. This is due to imbalance in the classes between the polyp and background in a colorectal polyp image, as the polyp regions occupy fewer pixels and most of the image is classified as background. A comparison between our method and the model in [30] shows that our method achieves a precision that is higher by 7.98%. Our proposed method was only outperformed by the network in [33]. Our method obtained slightly lower values for the mean IoU and dice coefficient of 92.3% and 95.99% as opposed to 96.95% and 98.45%, respectively. The reason for this could be that our method used a single network as our segmentation model, while the approach described in [33] used an ensemble method. In terms of network complexity, our proposed model has approximately 51.5 million trainable parameters, i.e., about half the number for the model in [33], yet our model performed only slightly worse, with differences of 4.65% and 2.46% in the mean IoU and dice coefficient, respectively. The concatenation of four dilated convolutions applied in parallel form introduced in our modified SegNet model serves the same purpose of aggregating multi-scale contextual information as the ensemble approach described in [33] but is accomplished in a single network. Overall, our proposed method yielded promising results, outperforming most of the existing CNN-based methods or giving comparable results. This meets the objectives of this research in terms of enhancing the performance of existing polyp segmentation models and reducing the model’s complexity by introducing three different modifications, i.e., skip connections, 5 × 5 convolutional filters, and concatenation of four dilated convolutions applied in parallel form on SegNet VGG-19.

## 5. Conclusions

In this paper, we have proposed a modified SegNet model, with VGG-19 as a framework, for automatic polyp segmentation. The main purpose of this model is to help medical experts diagnose polyps efficiently and to speed up the colon screening procedure. Three modifications were made to the original SegNet VGG-19, involving skip connections, 5 × 5 convolutional filters, and the concatenation of parallel dilated convolutions. We implemented and compared three modified SegNet models with different numbers of layers, and evaluated these on the CVC-ClinicDB, CVC-ColonDB, and ETIS-LaribPolypDB databases. From a total of 1187 images, 70% were randomly selected for training and the rest were used for testing. Our model obtained values of 96.06% for accuracy, 94.55% for sensitivity, 97.56% for specificity, 97.48% for precision, 92.3% for the mean IoU, and 95.99% for the Dice coefficient. Our modified SegNet model gave better or comparable results to those of previous models in the literature. In future work, we intend to embed the proposed model into a medical capsule robot and try it in a hospital setting with clinicians. We believe that this study will be useful in terms of the future development of CAD tools for polyp segmentation for the diagnosis and management of colorectal cancer.

## Figures and Tables

**Table 1:** Details of the publicly available datasets used for training and testing in this study.

Dataset	Images	Image Resolution (w × h)	Training Images	Testing Images
CVC-ClinicDB [34]	612 images from 29 video sequences	384 × 288	437	175
CVC-ColonDB [35]	379 images from 15 video sequences	574 × 500	260	119
ETIS-Larib Polyp DB [36]	196 images from 34 video sequences	1225 × 966	134	62

**Table 2:** Training times for the three modified SegNet models with different numbers of layers.

Network	100-Layer	154-Layer	208-Layer
Training time (mins)	331.16	355.52	475.19

**Table 3:** Polyp segmentation results from the testing dataset, for modified SegNet models with three different numbers of layers.

Network	Accuracy	Sensitivity	Specificity	Precision	Mean IoU	Dice Coefficient	F2 Score
100 layers	93.84%	89.86%	97.82%	97.63%	87.94%	93.58%	91.31%
154 layers	96.06%	94.55%	97.56%	97.48%	92.30%	95.99%	95.12%
208 layers	92.09%	87.63%	96.54%	96.20%	84.70%	91.72%	89.22%

**Table 4:** Segmentation accuracy of the three networks on the testing dataset, divided into six different accuracy ranges.

Testing Accuracy	Number of Testing Images		
	100-Layer Network	154-Layer Network	208-Layer Network
90–100%	286	304	272
80–89.99%	25	22	23
70–79.99%	20	9	27
60–69.99%	7	4	6
50–59.99%	9	10	13
0–49.99%	9	7	15

**Table 5:** Comparison of our modified SegNet model with the networks in [27,28] and the original SegNet VGG-19.

Author(s)	Method		IoU Polyp	IoU Background	Mean IoU
	Database	Segmentation			
[27]	CVC-ColonDB and CVC-ClinicDB	SegNet + LSTM	60.01%	94.54%	77.28%
[28]	CVC-ColonDB and CVC-ClinicDB	SegNet VGG-16	52.20%	93.30%	72.70%
Original SegNet	CVC-ColonDB, CVC-ClinicDB and ETIS-LaribPolypDB	SegNet VGG-19	67.23%	96.33%	85.79%
Proposed method	CVC-ColonDB, CVC-ClinicDB and ETIS-LaribPolypDB	Modified SegNet VGG-19	73.72%	97.10%	92.30%

**Table 6:** Comparison of our model with other recent convolutional neural network (CNN)-based methods.

Author(s)	Method		Accuracy	Sensitivity	Specificity	Precision	Mean IoU	Dice Coefficient
	Database	Segmentation						
[29]	CVC-ClinicDB and CVC-ColonDB	LinkNet	94.59%	80.23%	99.05%	88.65%	71.84%	79.07%
[30]	Affiliated Hospital of Hebei University, China	Mask-RCNN	-	-	-	89.50%	-	-
[31]	CVC-ColonDB and CVC-ClinicDB	Ensemble (Mask-RCNN)	-	76.25%	-	77.92%	69.46%	-
[32]	CVC-ClinicDB	Consecutive Deep Encoder–Decoder Network	98.70%	96.20%	94.20%	95.00%	-	89.10%
[33]	CVC-ClinicDB	Ensemble (U-Net, SegNet, and PSPNet)	-	-	-	-	96.16%	98.04%
	Cvc300		-	-	-	-	96.66%	98.30%
	ETIS-LaribPolypDB		-	-	-	-	96.95%	98.45%
Proposed method	CVC-ColonDB, CVC-ClinicDB and ETIS-LaribPolypDB	Modified SegNet VGG-19	96.06%	94.55%	97.56%	97.48%	92.30%	95.99%

**Figure A3:**
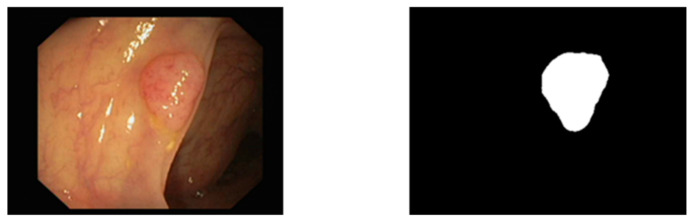
The image on the left is a raw colonoscopy image used for visualisation, and the image on the right represents the ground truth for the left image.

**Figure A4:**
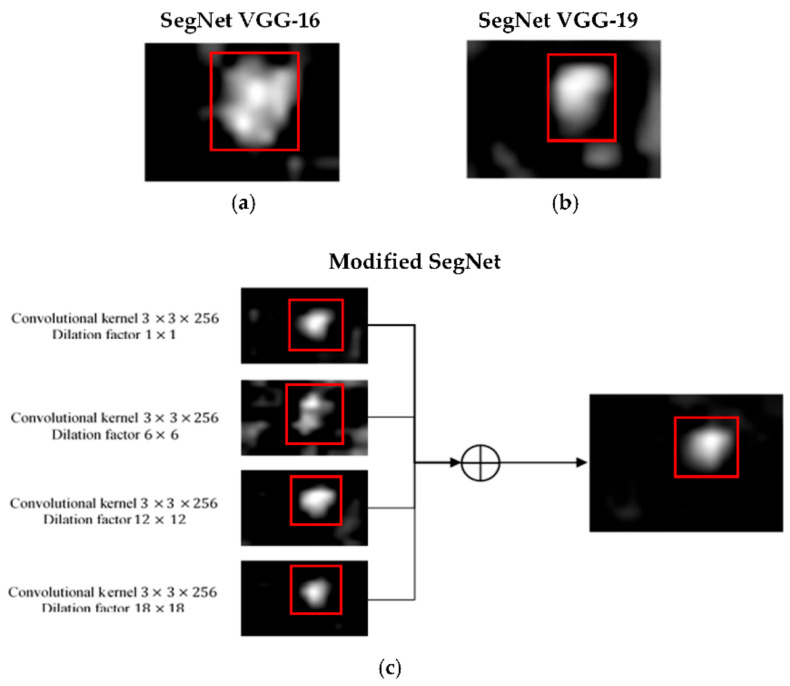
Intermediate sector between the encoder and decoder: (**a**) output activation map from the last max pooling layer in SegNet VGG-16; (**b**) output activation map from the last max pooling layer in SegNet VGG-19; (**c**) output activation maps from the ReLU layers from four different dilation factor convolution blocks (shown on the left), and the output of the ReLU layer from the convolution block after concatenation (shown on the right) for our modified SegNet network. The red boxes in the output activation maps indicate the location of polyps.

**Figure A5:**
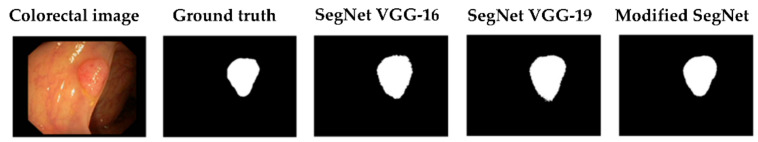
Segmentation results for a colorectal image from SegNet VGG-16, SegNet VGG-19, and our modified SegNet, with the ground truth for the colorectal image as a reference.

## Data Availability

Publicly available datasets were analyzed in this study. This data can be found here: [https://www.dropbox.com/s/p5qe9eotetjnbmq/CVC-ClinicDB.rar?dl=0], accessed on 1 July 2021.

## Appendix C

The activations of different SegNet layers can be displayed and visualised when a colonoscopy image is fed to the model. Each convolutional kernel can detect a specific feature, which can be presented in the form of activation. Through visualisation, the features learned by the network can be discovered by observing and comparing the activation areas with the original image. In our visualisation, we chose to examine only positive activations from the ReLU activation layer. White pixels in an activation map represent strong positive activations that are likely to be useful polyp features learned by the network. Black pixels represent the background, which does not include the object of interest (in this case a polyp). All three models (SegNet VGG-16, SegNet VGG-19, and our modified SegNet) are visualised in this section, and the results are compared separately for the encoder network, the intermediate sector between the encoder and decoder, the decoder network, and the final prediction map. Figure A3 shows the raw colonoscopy image used for visualisation purposes and its ground truth for reference.

### Appendix C.1. Visualisation of Activation in the Encoder Network

The final ReLU layer at each encoder depth was chosen to examine their activations and to visualise the encoder network. Visualisations of the encoder networks for all three SegNets are shown in Table A1. The red boxes in the output activation maps indicate the location of polyps.

**Table A1:** Activation visualisation in the final ReLU layer of all encoder depths for SegNet VGG-16, SegNet VGG-19, and our modified SegNet. The red boxes in the output activation maps indicate the location of polyps.

Encoder Network	Output Activation Map		
	SegNet VGG-16	SegNet VGG-19	Modified SegNet
1st encoder depth			
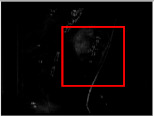
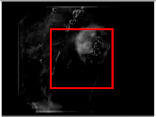
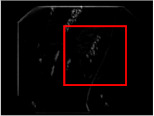
2nd encoder depth			
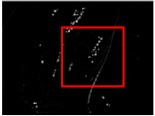
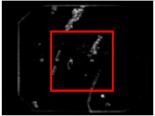
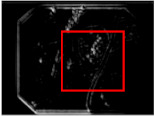
3rd encoder depth			
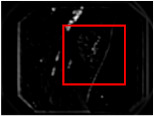
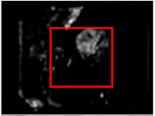
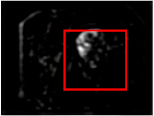
4th encoder depth			
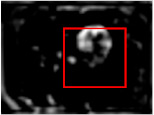
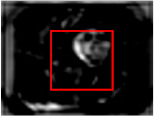
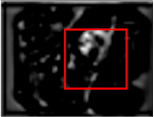
5th encoder depth			
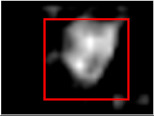
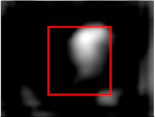
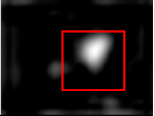

The results for the encoder networks for all the three networks were quite similar. However, the third encoder depth from SegNet VGG-19 and the modified SegNet activated more pixels in the polyp region than SegNet VGG-16. At the fifth encoder depth, SegNet VGG-16 could not correctly identify the edges of polyp, and the outer region was activated and classified as a polyp. SegNet VGG-19 and our modified SegNet significantly outperformed SegNet VGG-16, with more pixels and stronger activations in the polyp area. SegNet VGG-19 was unable to identify polyp edges as precisely as our modified SegNet due to a lack of information about the polyp edges, leading to a discontinuity in polyp edge detection and an over-activated region. The modified SegNet model was shown to be capable of capturing more stable and better continuity features for the polyp edges as a whole. The resulting activation visualisation shows that the strongest white pixel activations were accurately determined to be within the polyp area.

### Appendix C.2. Visualisation of Activation in the Intermediate Sector between Encoder and Decoder

The purpose of studying the intermediate sector between the encoder and decoder is to determine the beneficial effect on the network of concatenating of four dilated convolutions in parallel form with different dilation factors in our modified SegNet compared to the original SegNet VGG-16 and SegNet VGG-19 models. A comparison of these visualisations is shown in Figure A4.

There are no structural differences in the intermediate sector between the encoder and decoder or the output of the fifth encoder depth for the SegNet VGG-16 and SegNet VGG-19. The output of the last max pooling layer of the encoder network was fed directly into the corresponding max unpooling layer. In our modified SegNet, the four outputs generated from the four convolutions with different dilated factors were concatenated at the transition between the encoder and decoder networks. An ordinary convolution block was applied after four parallel dilated convolutions to concatenate them together. Figure A4a,b show visualisations of the output activation maps from the max pooling layer at the end of the encoder network for SegNet VGG-16 and SegNet VGG-19, respectively. Figure A4c shows the visualisation of ReLU layers from the four dilated convolution blocks, and the convolution block after concatenation.

As shown in Figure A4c, different dilation factor of convolution blocks viewed the polyp image in different resolutions, and consequently gave different output activation maps. Convolution blocks with dilation factors of 1 × 1, 12 × 12, and 18 × 18 were capable of recognising the polyp and giving the strongest pixel activation within the correct location. The only unexpected outcome was that the convolution block with a dilation factor of 6 × 6 performed exceptionally poorly in terms of discriminating between the polyp and the background. Nevertheless, the new output activation map generated from the convolution block after concatenation contained significantly less noise compared to the output activation at the fifth encoder depth.

### Appendix C.3. Visualisation of Activation in the Decoder Network and Final Prediction Map

For the decoder network, we compared the output activation maps from the max unpooling layer and the first ReLU layer at each decoder depth for the three networks (SegNet VGG-16, SegNet VGG-19, and the modified SegNet). In this section, we also visualise the skip connection and the addition layer in the modified SegNet to analyse their beneficial effects on the segmentation results. Output activation maps from the three networks are shown in Table A2. The red boxes in the output activation maps indicate the location of polyps.

**Table A2:** Activation visualisation for the max unpooling and ReLU layers at the beginning of all decoder depths, for SegNet VGG-16, SegNet VGG-19, and the modified SegNet, and the skip connection and addition layers at all decoder depths for the modified SegNet. The red boxes in the output activation maps indicate the location of polyps.

Decoder Network	Output Activation Map			
	SegNet VGG-16		SegNet VGG-19	
	Max Unpooling Layer	ReLU Layer	Max Unpooling Layer	ReLU Layer
1st encoder depth				
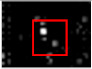
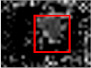
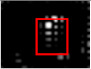
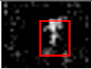
2nd encoder depth				
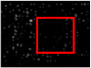
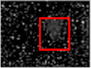
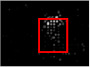
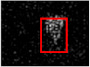
3rd encoder depth				
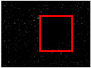
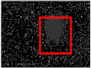
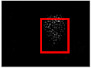
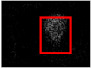
4th encoder depth				
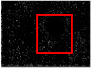
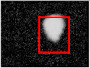
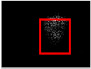
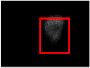
5th encoder depth				
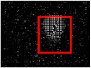
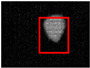
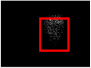
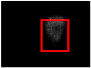
**Decoder network**	**Modified SegNet**			
	**Max unpooling + skip connection = addition**			**ReLU layer**
1st encoder depth	+ =			
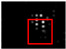
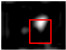
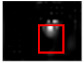
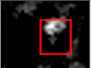
2nd encoder depth	+ =			
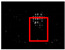
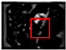
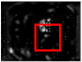
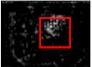
3rd encoder depth	+ =			
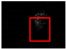
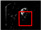
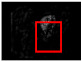
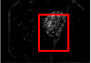
4th encoder depth	+ =			
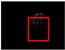
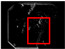
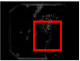
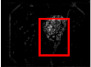
5th encoder depth	+ =			
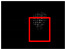
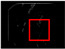
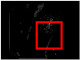
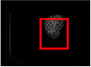

From Table A2, it can be seen that SegNet VGG-16 produced the most noise and disturbance in the activation maps at all decoder depths. A large number of non-polyp pixels were activated, which tended to degrade the segmentation results. This model performed the worst of all three networks. SegNet VGG-19 was better in terms of recognising polyps than SegNet VGG-16, due to its deeper network structure. As can be seen from Table A2, many of the pixels activated in the output maps from the max unpooling and ReLU layers in the SegNet VGG-16 decoder network were highly scattered. The output activation maps from the max unpooling and ReLU layers of the SegNet VGG-19 decoder network were better, as most of the strongly activated pixels were located within the polyp region, particularly from the third decoder depth onwards.

The modified SegNet performed slightly better than SegNet VGG-19. It can be observed from Table A2 that the output activation maps from the max unpooling layers at every decoder depth of the modified SegNet contained less noise, and were more accurate and more centralised on the polyp region compared to SegNet VGG-19. This was a result of the inclusion of the skip connection architecture, which allows some of the information lost throughout the network to be restored. When the third decoder depth is compared, it can be seen that the noise in the output activation maps from the ReLU layers of the modified SegNet and SegNet VGG-19 is different. SegNet VGG-19 shows a greater distortion in the polyp information and noise that is scattered, as shown in dotted form. In contrast, the information added from the encoder path allowed our modified SegNet to give a more complete representation of the polyp features and noise in the colon environment. We can see that features such as the texture, blood vessels, and the change in intensity at the edge of protrusions, folds, and bends were better extracted by the modified SegNet, as the pixel activation was more continuous than in SegNet VGG-19. Although the image information fed into the decoder network via the skip connections contained background noise, the pixel activation, especially in the polyp region, could be maximised to eliminate this noise, thus helping the network to better discriminate and segment out the polyp from the background. We can conclude that the skip connections and addition layers are effective in improving a CNN, and particularly the SegNet visualised here. The reduced over-segmentation in the results of the modified SegNet is shown even more clearly in Figure A5, which gives a comparison of the segmentation results from all three networks.

Although all three networks managed to segment out the correct polyp location and region, SegNet VGG-16 yielded the roughest results. Some over-segmented regions were present at the edge of the polyp in the results from both SegNet VGG-16 and SegNet VGG-19. Our modified SegNet achieved the most promising segmentation outcome of the three networks, with the smoothest outline and the most similar binary mask to the ground truth reference.

## Appendix D

$$Accuracy=\;\frac{TN+TP}{TN+TP+FN+FP}$$

$$Sensitivity=\;\frac{TP}{TP+FN}$$

$$Specificity=\;\frac{TN}{TN+FP}$$

$$Precision=\;\frac{TP}{TP+FP}$$

$$Mean\;IoU=\;\frac{TP}{TP+FP+FN}$$

$$Dice\;Coefficient=\;\frac{2TP}{2TP+FP+FN}$$

$$F2\;score=\;5\times \frac{Precision\;\times Sensitivity}{4\times Precision+Sensitivity}$$
